# Peroxiredoxin-5 Knockdown Accelerates Pressure Overload-Induced Cardiac Hypertrophy in Mice

**DOI:** 10.1155/2022/5067544

**Published:** 2022-01-29

**Authors:** Chengyun Hu, Feibiao Dai, Jiawu Wang, Lai Jiang, Di Wang, Jie Gao, Jun Huang, Jianfeng Luo, Fei Tang, Zhetao Zhang, Chaoliang Tang

**Affiliations:** ^1^Department of Anesthesiology, The First Affiliated Hospital of USTC, Division of Life Sciences and Medicine, University of Science and Technology of China, Hefei, Anhui 230001, China; ^2^Department of Anesthesia, Wannan Medical College, Wuhu, Anhui 241002, China; ^3^Department of Obstetrics and Gynecology, The First Affiliated Hospital of USTC, Division of Life Sciences and Medicine, University of Science and Technology of China, Hefei, Anhui 230001, China; ^4^Department of Anesthesiology, The Second Affiliated Hospital of Anhui Medical University, Hefei, Anhui 230601, China; ^5^Department of Anesthesiology, The First Affiliated Hospital of Anhui Medical University, Hefei, Anhui 230022, China; ^6^Department of Anesthesiology, The People's Hospital of Chizhou, Chizhou, Anhui 247000, China; ^7^Department of Cardiology, The First Affiliated Hospital of USTC, Division of Life Sciences and Medicine, University of Science and Technology of China, Hefei, Anhui 230001, China; ^8^Department of Pharmacy, The First Affiliated Hospital of USTC, Division of Life Sciences and Medicine, University of Science and Technology of China, Hefei, Anhui 230036, China

## Abstract

A recent study showed that peroxiredoxins (Prxs) play an important role in the development of pathological cardiac hypertrophy. However, the involvement of Prx5 in cardiac hypertrophy remains unclear. Therefore, this study is aimed at investigating the role and mechanisms of Prx5 in pathological cardiac hypertrophy and dysfunction. Transverse aortic constriction (TAC) surgery was performed to establish a pressure overload-induced cardiac hypertrophy model. In this study, we found that Prx5 expression was upregulated in hypertrophic hearts and cardiomyocytes. In addition, Prx5 knockdown accelerated pressure overload-induced cardiac hypertrophy and dysfunction in mice by activating oxidative stress and cardiomyocyte apoptosis. Importantly, heart deterioration caused by Prx5 knockdown was related to mitogen-activated protein kinase (MAPK) pathway activation. These findings suggest that Prx5 could be a novel target for treating cardiac hypertrophy and heart failure.

## 1. Introduction

Pathological cardiac hypertrophy is a common pathophysiological process of various cardiovascular diseases, including hypertension, myocardial infarction, and heart failure [1–3]. It presents as thickening of the ventricular wall and decreased compliance of the ventricular wall [4–6]. Multiple mechanisms have been identified in the regulation of pathological cardiac hypertrophy, including oxidative stress, inflammation, autophagy, and cardiomyocyte apoptosis [7–9]. Thus, targeting molecules or genes associated with the above processes is crucial for the treatment of pathological cardiac hypertrophy.

Peroxiredoxins (Prxs) are a superfamily of antioxidant peroxidases that scavenge hydrogen peroxide (H_2_O_2_) and alkyl hydroperoxides [10–13]. At present, Prxs are identified as important regulators of redox homeostasis and participate in a series of cell functions. Many studies have shown that some family members of Prxs play an important role in the development of pathological cardiac hypertrophy [14, 15]. Cardiomyocyte-specific Prx1 overexpression in mice attenuates cardiac hypertrophy and dysfunction under pressure overload [14]. Similarly, overexpression of Prx3 prevents cardiac hypertrophy and failure after myocardial infarction in mice [15]. As an important member of the Prx family, Prx5 has antioxidant protective functions and can effectively remove reactive oxygen species (ROS) [16, 17]. However, the involvement of Prx5 in cardiac hypertrophy remains unclear.

In this study, we explored the role and mechanisms of Prx5 in pathological cardiac hypertrophy and dysfunction. We confirmed that Prx5 knockdown accelerates pressure overload-induced cardiac hypertrophy and dysfunction in mice by activating oxidative stress and cardiomyocyte apoptosis. Importantly, heart deterioration caused by Prx5 knockdown was related to mitogen-activated protein kinase (MAPK) activation. These findings suggest that Prx5 could be a novel target for treating cardiac hypertrophy and heart failure.

## 2. Materials and Methods

### 2.1. Animals and Animal Model

All animal procedures in this study were approved by the Animal Care and Use Committee of Anhui Medical University. Male C57BL/6 mice (8-10 weeks, 23-26 g) were purchased from Beijing HFK Bioscience Co., Ltd. (Beijing, China) and were housed in a well-ventilated environment. Transverse aortic constriction (TAC) surgery was performed to establish pressure overload-induced cardiac hypertrophy model as previously described [18]. Two weeks before TAC surgery, mice received a heart injection of AAV9-shPrx5 (1 × 10^11^ viral particles/mouse) to knockdown Prx5 in the myocardium. Four weeks after TAC or the sham procedure, mice were euthanized by intraperitoneal injection of 200 mg/kg pentobarbital sodium. Then, the hearts, lungs, and tibia were harvested and measured to calculate the heart weight/body weight (HW/BW, mg/g), lung weight to body weight (LW/BW, mg/g), heart weight to tibia length (HW/TL, mg/mm), and lung weight/tibia length (LW/TL, mg/mm) ratios in the mice.

### 2.2. Echocardiography Analysis

Cardiac function was detected using a Vivid 7000 ultrasound equipped with a 14 MHz transducer. In short, the mice were anesthetized, and the left ventricle (LV) geometry was assessed in both parasternal long-axis and short-axis views. The heart rate (HR), LV internal diameter at end-diastole (LVIDd), LV internal diameter at end-systole (LVIDs), LV posterior wall thickness of end systolic (LVPWs), LV posterior wall thickness of end diastolic (LVPWd), interventricular septal thickness at end-diastole (IVSd), interventricular septal thickness at end-systole (IVSs), and LV fractional shortening (LVFS) were determined.

### 2.3. Histological and TUNEL Analysis

The mice were sacrificed immediately after echocardiography measurements, and the hearts were harvested and then placed in 4% paraformaldehyde. Then, the heart sections were prepared and stained with hematoxylin and eosin (HE) and wheat germ agglutinin (WGA) for morphological analyses and evaluation of the cross-sectional area (CSA). In addition, heart sections were stained with picrosirius red (PSR) to assess collagen deposition. To detect cardiomyocyte apoptosis, TUNEL staining was performed as described in our previous study. The sections were visualized using microscopy, and all images were analyzed using Image-Pro Plus 6.0.

### 2.4. Neonatal Rat Cardiomyocyte (NRCM) Culture and Treatment

Primary neonatal rat cardiomyocytes (NRCMs) were isolated from 1- to 2-day-old Sprague-Dawley rats as previously described [19]. Then, the NRCMs were cultured in plating medium consisting of DMEM/F12 containing 15% fetal bovine serum (FBS), 0.1 mM BrdU, and 100 mg/mL penicillin/streptomycin. To knockdown Prx5 in vitro, Prx5 siRNA was used according to manufacturer's instructions. Then, the NRCMs were stimulated with angiotensin II (Ang II, 1 *μ*M) for 48 h.

### 2.5. Immunofluorescence Analysis

The NRCMs were fixed with 4% paraformaldehyde and permeabilized with 0.2% Triton X-100. Then, the NRCMs were stained with *α*-actinin and the indicated fluorescent secondary antibody and then stained with DAPI. Finally, the NRCMs were visualized under a fluorescence microscope, and all images were analyzed using Image-pro Plus 6.0.

### 2.6. Quantitative Real-Time PCR

Total mRNA was extracted from ventricular tissue and NRCMs and then converted to cDNA using the RNA PCR Kit). PCR amplification was performed and quantified using an ABI PRISM 7000 Sequence Detection System. The relative mRNA expression levels of target genes were analyzed and normalized to the mRNA expression level of glyceraldehyde-3-phosphate dehydrogenase (GAPDH). The sequences of the primers used are presented in Table 1.

### 2.7. Western Blotting

Protein lysates of ventricular tissue and NRCMs were prepared and the protein concentrations were then measured. The proteins were loaded and run on SDS-PAGE and transferred to a PVDF membrane. The PVDF membranes were subsequently blocked with 5% PVDF and incubated with primary antibodies against Prx5, GAPDH, Bax, Bcl-2, ERK, p-ERK, JNK, p-JNK, p38, and p-p38. After washing, the PVDF membranes were incubated with a secondary antibody and visualized with an infrared imaging system according to manufacturer's protocol. The specific protein expression levels were normalized to that of GAPDH.

### 2.8. Measurement of Oxidative Stress Level

Dihydroethidium (DHE) staining was performed according to manufacturer's protocol. In short, frozen sections of ventricular tissue were incubated with 10 *μ*M DHE in PBS in a humidified and light-protected chamber. The images were then taken with a laser microscope and analyzed using Image-Pro Plus 6.0. In addition, superoxide dismutase (SOD), glutathione (GSH), malondialdehyde (MDA), and H_2_O_2_ in LV tissue or NRCMs were detected using kits purchased from Beyotime Biotechnology Corporation (China).

### 2.9. Statistical Analysis

The data are expressed as the mean ± standard deviation. Comparisons between two groups were analyzed using an unpaired Student's *t* test. Differences among multiple groups were assessed using an analysis of variance followed by one-way analysis of variance. A value of *P* < 0.05 was considered statistically significant.

## 3. Results

### 3.1. Prx5 Expression Is Increased in Hypertrophic Hearts and Isolated NRCMs

**Figure 1:**
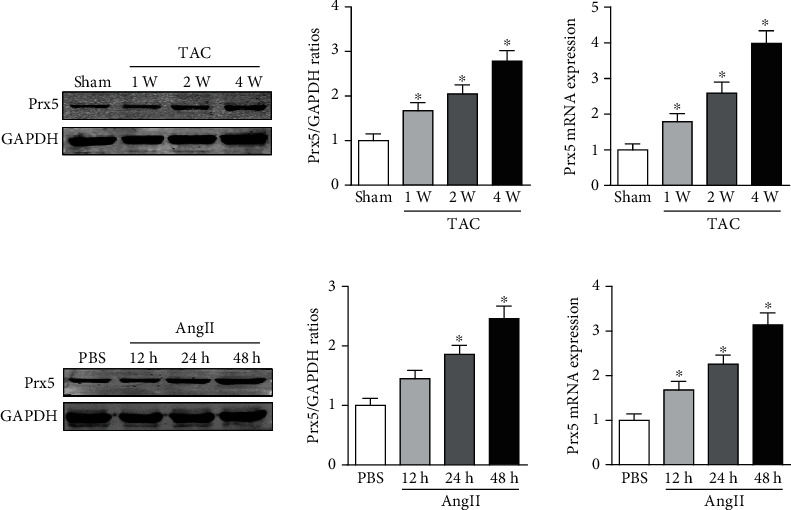
Prx5 expression is increased in hypertrophic hearts and isolated NRCMs. (a) The expression of Prx5 in heart tissues was measured by Western blotting and RT-PCR (*n* = 4, ^∗^*P* < 0.05 vs. the sham group). (c) The expression of Prx5 in heart tissues was measured by Western blotting and RT-PCR (*n* = 4, ^∗^*P* < 0.05 vs. the PBS group).

The results showed that Prx5 expression was gradually upregulated in the hearts of mice subjected to TAC surgery (Figure 1(a)). In accordance with this, higher Prx5 levels were also detected in isolated NRCMs after Ang II stimulation (Figure 1(b)). Together, these data suggest that Prx5 may participate in the development of cardiac hypertrophy.

### 3.2. Prx5 Knockdown Accelerates Pressure Overload-Induced Cardiac Dysfunction

After TAC surgery, animals exhibited LV dilatation and thickening, as indicated by increased LVIDd, LVIDs, LVPWd, LVPWs, IVSd, and IVSs and decreased FS. However, Prx5 knockdown further aggravated pressure overload-induced cardiac dysfunction (Table 2). In addition, there were no significant differences in HR among the four groups.

### 3.3. Prx5 Knockdown Accelerates Pressure Overload-Induced Cardiac Hypertrophy

**Figure 2:**
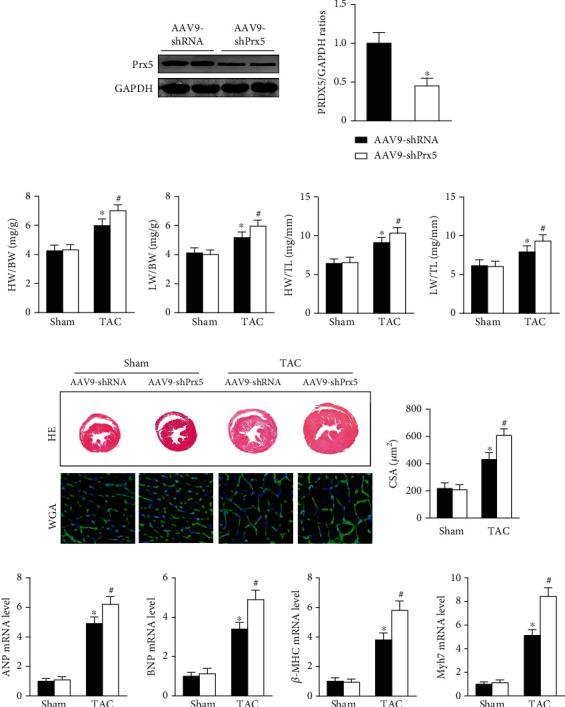
Prx5 knockdown accelerates pressure overload-induced cardiac hypertrophy. (a) The levels of Prx5 after injection with AAV9-shPrx5 (*n* = 4). (b) Results for the HW/BW ratio, HW/TL ratio, LW/BW ratio, LW/TL ratio, and CSA of each group (*n* = 6). (c) HE and WGA staining were performed in each group (*n* = 6; scale bar, 50 *μ*m). (d) The expression of ANP, BNP, *β*-MHC, and Myh7 was measured by RT-PCR in each group (*n* = 5). ^∗^*P* < 0.05 vs. the sham group; ^#^*P* < 0.05 vs. the TAC group.

As shown in Figure 2, AAV9-shPrx5 caused decreased expression of Prx5 in hearts (Figure 2(a)). Four weeks after TAC surgery, Prx5 knockdown accelerated pressure overload-induced cardiac hypertrophy, as evidenced by increased HW/BW, HW/TL, LW/BW, and LW/TL ratios and increased CSA (Figures 2(b) and 2(c)). In addition, higher mRNA levels of atrial natriuretic peptide (ANP), brain natriuretic peptide (BNP), *β*-myosin heavy chain (*β*-MHC), and Myosin Heavy Chain 7 (Myh7) were also found in the Prx5 knockdown group after TAC surgery (Figure 2(c)).

### 3.4. Prx5 Knockdown Accelerates Pressure Overload-Induced Cardiac Fibrosis

**Figure 3:**
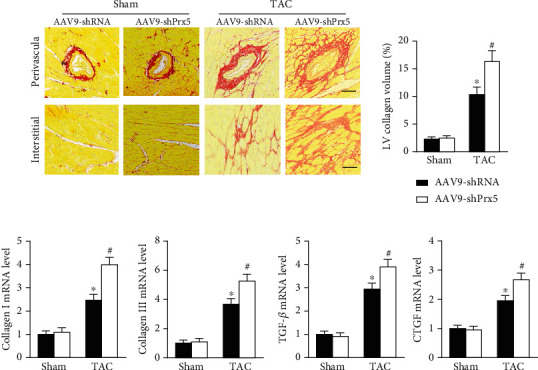
Prx5 knockdown accelerates pressure overload-induced cardiac fibrosis. (a) PSR staining was performed in each group (*n* = 6; scale bar, 50 *μ*m). (b) The expression of collagen I, collagen III, TGF-*β*, and CTGF was measured by RT-PCR in each group (*n* = 6). ^∗^*P* < 0.05 vs. the sham group; ^#^*P* < 0.05 vs. the TAC group.

As shown in Figure 3, dramatic collagen deposition was observed in the mice after TAC surgery and was further increased in the Prx5 knockdown group (Figure 3(a)). Similarly, after TAC surgery, the mRNA expression levels of several fibrosis markers, including collagen I, collagen III, transforming growth factor (TGF)-*β*, and connective tissue growth factor (CTGF), were also further increased in the Prx5 knockdown group (Figure 3(b)).

### 3.5. Prx5 Knockdown Accelerates Pressure Overload-Induced Oxidative Stress and Apoptosis in Mice

**Figure 4:**
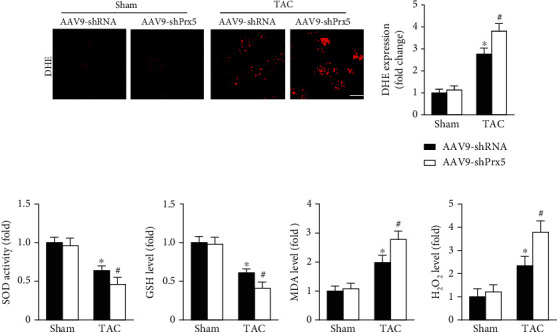
Prx5 knockdown accelerates pressure overload-induced oxidative stress and apoptosis. (a) DHE staining was performed in each group (*n* = 5; scale bar, 100 *μ*m). (b) Quantitative results of SOD activity and GSH, MDA, and H_2_O_2_ levels in the hearts of each group (*n* = 6). ^∗^*P* < 0.05 vs. the sham group; ^#^*P* < 0.05 vs. the TAC group.

DHE staining was used to evaluate in vivo oxidative stress levels. The results showed that the oxidative stress level was dramatically increased in the Prx5 knockdown group after TAC surgery (Figure 4(a)). Moreover, Prx5 knockdown markedly decreased SOD activity and GSH levels and increased MDA and H_2_O_2_ levels in TAC-treated mice (Figure 4(b)).

### 3.6. Prx5 Knockdown Accelerates Pressure Overload-Induced Apoptosis in Mice

**Figure 5:**
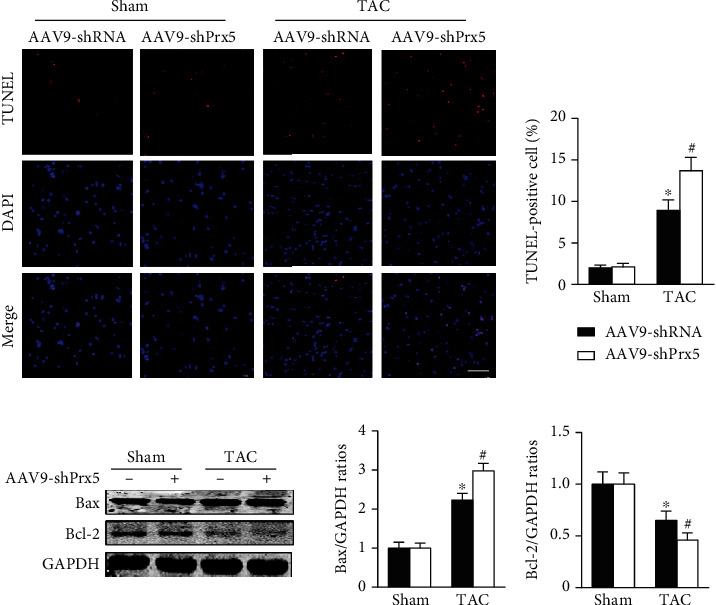
Prx5 knockdown accelerates pressure overload-induced apoptosis in mice. (a) TUNEL staining was performed in each group (*n* = 4; scale bar, 50 *μ*m). (b) The expression of Bax and Bcl-2 was measured by Western blot in each group (*n* = 4). ^∗^*P* < 0.05 vs. the sham group; ^#^*P* < 0.05 vs. the TAC group.

TUNEL staining was used to evaluate apoptosis levels in the heart. The results showed that the number of TUNEL-positive cells was dramatically increased in the Prx5 knockdown group after TAC surgery (Figure 5(a)). Moreover, Prx5 knockdown increased Bax and decreased Bcl-2 levels in TAC-treated mice (Figure 5(b)).

### 3.7. Prx5 Knockdown Accelerates AngII-Induced Cardiomyocyte Hypertrophy In Vitro

**Figure 6:**
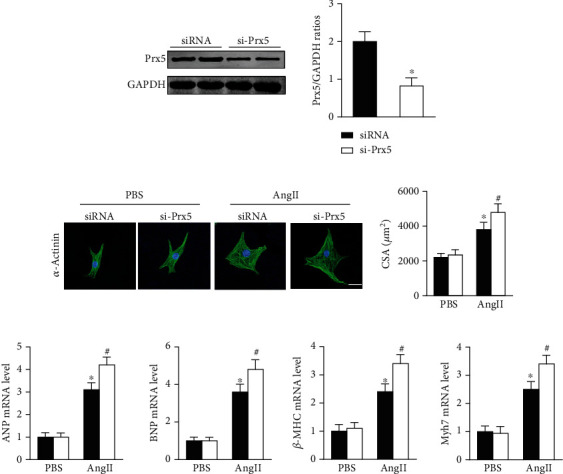
Prx5 knockdown accelerates AngII-induced cardiomyocyte hypertrophy in vitro. (a) The levels of Prx5 were measured by Western blot (*n* = 4). (b) Immunofluorescence staining for *α*-actinin was performed in each group (*n* = 4; scale bar, 25 *μ*m). (c) The expression of ANP, BNP, *β*-MHC, and Myh7 was measured by RT-PCR in each group (*n* = 6). ^∗^*P* < 0.05 vs. the PBS group; ^#^*P* < 0.05 vs. the AngII group.

Consistent with the in vivo results, si-Prx5 led to decreased expression of Prx5 in NRCMs (Figure 6(a)). After 48 h of AngII stimulation, the NRCMs exhibited clear hypertrophy by augmentation in CSA and increased mRNA levels of ANP, BNP, *β*-MHC, and Myh7 (Figures 6(b) and 6(c)). Interestingly, Prx5 knockdown accelerated AngII-induced cardiomyocyte hypertrophy in vitro (Figures 6(b) and 6(c)).

### 3.8. Prx5 Knockdown Accelerates AngII-Induced Oxidative Stress and Apoptosis In Vitro

**Figure 7:**
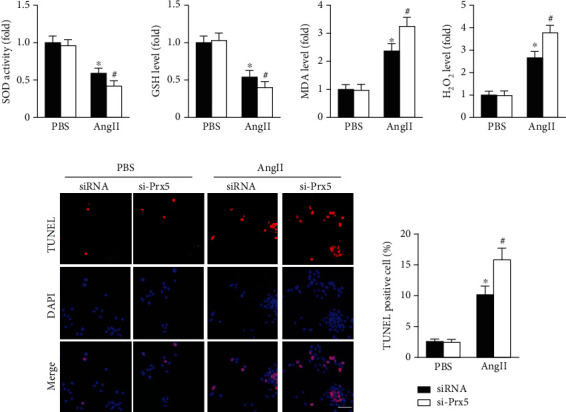
Prx5 knockdown accelerates AngII-induced oxidative stress and apoptosis in vitro. (a) Quantitative results of SOD activity and GSH, MDA, and H_2_O_2_ levels in the hearts of each group (*n* = 6). (c) TUNEL staining was performed in each group (*n* = 4; scale bar, 50 *μ*m). ^∗^*P* < 0.05 vs. the PBS group; ^#^*P* < 0.05 vs. the AngII group.

The results showed that AngII treatment markedly decreased SOD activity and GSH levels and increased MDA and H_2_O_2_ levels in vitro, while these effects were further augmented by Prx5 knockdown (Figure 7(a)). TUNEL staining further showed that Prx5 knockdown further increased the number of TUNEL-positive cells in vitro (Figure 7(b)).

### 3.9. Effect of Prx5 on the MAPK Signaling Pathway

**Figure 8:**
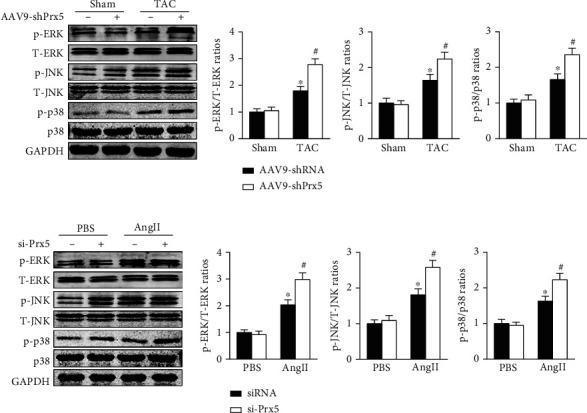
Effect of Prx5 on the MAPK signaling pathway. (a) The expression of p-ERK, t-ERK, p-JNK, t-JNK, p-p38, and p38 in the heart was measured by Western blotting (*n* = 4). (b) The expression of p-ERK, ERK, p-JNK, JNK, p-p38, and p38 in NVCMs was measured by Western blotting (*n* = 4). ^∗^*P* < 0.05 vs. the sham or PBS group; ^#^*P* < 0.05 vs. the TAC or AngII group.

Previous research has widely implicated MAPK signaling in cardiac hypertrophy. Thus, we investigated whether the effects of Prx5 are associated with the MAPK signaling pathway. The results showed that the phosphorylated levels of ERK, p38, and JNK were significantly increased in TAC-treated mice. However, these effects were further augmented by Prx5 knockdown (Figure 8(a)). Consistent with the in vivo results, Prx5 knockdown also increased the phosphorylation levels of ERK, p38, and JNK in NRCMs after AngII treatment (Figure 8(b)).

## 4. Discussion

In the present study, we uncovered a novel role of Prx5 in pressure overload-induced cardiac hypertrophy. The results showed that the mRNA and protein expression of Prx5 were noticeably upregulated in hypertrophic hearts and AngII-stimulated cardiomyocytes. In addition, Prx5 knockdown accelerated pressure overload-induced cardiac hypertrophy and dysfunction in mice by activating oxidative stress and cardiomyocyte apoptosis. Importantly, heart deterioration caused by Prx5 knockdown was related to MAPK activation. These findings suggest that Prx5 could be a novel target for treating cardiac hypertrophy and heart failure.

Prx5, also called PrxV/AOEB166/PMP20/PLP/ACR1, was discovered twenty years ago and is widely expressed in mammalian tissues [20]. As an important member of the Prxs family, Prx5 plays a central role in redox signal transduction and exhibits high scavenging activity toward oxidative stress [16] [21]. Previous research has shown that Prx5 exhibits a protective role in a variety of diseases, including brain lesions, aging, obesity, and cancer [22–24]. Recombinant Prx5 administration provided protection against N-methyl-D-aspartate-mediated brain lesions and neuronal death in newborn mice [22]. In an obesity model induced by a high-fat diet, deletion of Prx5 increased susceptibility to obesity and adipogenesis by increasing ROS generation and adipogenic gene expression [23]. However, little is known about the exact role and mechanisms of Prx5 in the development of cardiac hypertrophy and dysfunction. In the present study, we first confirmed that Prx5 was upregulated in hypertrophic mouse hearts and AngII-stimulated NRCMs, indicating that Prx5 might be involved in the progression and development of pathological cardiac hypertrophy. In addition, AAV9-shPrx5 was used to knock down Prx5 in the myocardium. The results showed that Prx5 knockdown accelerates pressure overload-induced cardiac dysfunction, hypertrophy, and fibrosis in mice. Consistent with the in vivo results, Prx5 knockdown also accelerated AngII-induced cardiomyocyte hypertrophy in vitro.

Oxidative stress is described as a common pathological feature of various cardiovascular diseases [25–28]. As a natural byproduct of the metabolic utilization of oxygen, ROS are oxygen-containing molecules with highly reactive properties and represent crucial drivers of oxidative stress [29–31]. Under pathological conditions of pressure overload, excessive ROS result in cardiomyocyte death or functional damage and ultimately cardiac dysfunction [32–34]. There is likely benefit from the suppression of oxidative stress and countering excessive production of ROS in pathological cardiac hypertrophy therapy.

Many studies have shown that Prx5 has antioxidant protective functions and can effectively remove oxidative stress [17] [23]. Thus, we investigated whether Prx5 is involved in the occurrence of cardiac hypertrophy and dysfunction by regulating oxidative stress. The results showed that Prx5 knockdown markedly decreased SOD activity and GSH levels and increased MDA and H_2_O_2_ levels in TAC-treated mice. DHE staining results also showed that the oxidative stress level was dramatically increased in the Prx5 knockdown group after TAC surgery. Consistent with the in vivo results, Prx5 knockdown accelerated AngII-induced oxidative stress in vitro. These results indicate that the deterioration effect of Prx5 knockdown is related to oxidative stress.

Apoptosis is known to contribute to various cardiovascular diseases, including heart failure, myocardial infarct, and reperfusion injury [35, 36]. Previous research has also shown that cardiac hypertrophy is related to a reduced cell number due to enhanced apoptosis [37, 38]. In addition, oxidative stress has been shown to be responsible for cardiomyocyte apoptosis [39–41]. Thus, we asked whether Prx5 affects cardiomyocyte apoptosis in pathological cardiac hypertrophy. The results showed that the expression of Bax was upregulated, the expression of Bcl-2 was lower, and there were more TUNEL-positive cells in the TAC group than in the sham group. These effects were further augmented by Prx5 knockdown. In addition, the results further confirmed the in vitro cell experiments, indicating that the deterioration effect of Prx5 knockdown is associated with cardiomyocyte apoptosis.

As intracellular signaling proteins, MAPKs have been shown to regulate various cellular processes, including cell size, cell growth, and cell survival, in response to extracellular stimuli [41–43]. It is well established that the activation of MAPK signaling increases cardiac damage and exacerbates pathological cardiac hypertrophy [44–46]. In this study, Prx5 knockdown accelerated pressure overload-induced cardiac hypertrophy and dysfunction. However, the role of Prx5 in MAPK signaling activation in pathological cardiac hypertrophy was unclear. Thus, we examined the phosphorylation and total expression of ERK/JNK/p38 in hypertrophic hearts and AngII-stimulated cardiomyocytes. The results showed that Prx5 knockdown significantly induced the phosphorylation of ERK/JNK/p38 in the TAC group, but the expression of total ERK/JNK/p38 remained unchanged. These results indicate that the deterioration effect of Prx5 knockdown is associated with activation of MAPK signaling.

### 4.1. Clinical Significance

Pathological cardiac hypertrophy is a common pathophysiological process of various cardiovascular diseases, including hypertension, myocardial infarction, and heart failure. Currently, there is no specific treatment to effectively reverse cardiac pathological hypertrophy and reduce the morbidity and mortality of heart failure. In this study, we demonstrated that Prx5 knockdown accelerated pressure overload-induced cardiac hypertrophy and dysfunction in mice by activating oxidative stress and cardiomyocyte apoptosis. Importantly, heart deterioration caused by Prx5 knockdown was related to MAPK activation. These findings provided a new target for the prevention and treatment of cardiac hypertrophy and heart failure.

### 4.2. Study Limitations

This study was subject to the following limitations. First, as pathological cardiac hypertrophy is a multifactorial syndrome, we cannot exclude the possibility that Prx5 utilizes other pathways to protect the heart under pressure overload. Thus, more research is needed to determine the mechanism underlying the cardioprotective effects of Prx5. In addition, in our study, mice received a heart injection of AAV9-shPrx5 to knock down Prx5 in the myocardium. However, animals with cardiac-specific overexpression or knockout of Prx5 may more precisely demonstrate the important function of Prx5 in pathological cardiac hypertrophy and dysfunction.

Taken together, our results have uncovered novel insights into the regulation of pathological cardiac hypertrophy and dysfunction by Prx5. The results showed that Prx5 knockdown accelerates pressure overload-induced cardiac hypertrophy and dysfunction. Our data indicate that Prx5 may be an attractive target for the prevention and treatment of pathological cardiac hypertrophy and heart failure.

## Figures and Tables

**Table 1:** Primer sequences for RT-PCR assays.

Gene	Species	Sequence (5′-3′)	
PRDX5	Mouse	Forward	GCTGCAAAGCCAGTTCTGTG
		Reverse	CCACTGAGGGAATGGCATCTC
PRDX5	Rat	Forward	GCAAGGTTCAGCTCCTGGCT
		Reverse	CAGGTGAGGCCTGTGCCATC
ANP	Mouse	Forward	CCTGTGTACAGTGCGGTGTC
		Reverse	AAGCTGTTGCAGCCTAGTCC
ANP	Rat	Forward	AAAGCAAACTGAGGGCTCTGCTCG
		Reverse	TTCGGTACCGGAAGCTGTTGCA
BNP	Mouse	Forward	CTCAAGCTGCTTTGGGCACAAGAT
		Reverse	AGCCAGGAGGTCTTCCTACAACAA
BNP	Rat	Forward	CAGCAGCTTCTGCATCGTGGAT
		Reverse	TTCCTTAATCTGTCGCCGCTGG
*β*-MHC	Mouse	Forward	TCTACCCAGCCAAGATCAAAGT
		Reverse	CCCATTCCTAATAAGCTGTGTGG
*β*-MHC	Rat	Forward	TCTGGACAGCTCCCCATTCT
		Reverse	CAAGGCTAACCTGGAGAAGATG
Myh7	Mouse	Forward	ACTGTCAACACTAAGAGGGTCA
		Reverse	TTGGATGATTTGATCTTCCAGGG
Myh7	Rat	Forward	TGCTGTTATTGCTGCCATTG
		Reverse	AGGAGTTATCATTCCGAACTGTC
TGF-*β*	Mouse	Forward	TGCTTCAGCTCCACAGAGAA
		Reverse	TGGTTGTAGAGGGCAAGGAC
TGF-*β*	Rat	Forward	ATTCCTGGCGTTACCTTGG
		Reverse	AGCCCTGTATTCCGTCTCCT
CTGF	Mouse	Forward	TGTGTGATGAGCCCAAGGAC
		Reverse	AGTTGGCTCGCATCATAGTTG
CTGF	Rat	Forward	ACACAAGGGTCTCTTCTGCG
		Reverse	ACAGGGTGCACCATCTTTGG
Collagen I	Mouse	Forward	TGGTACATCAGCCCGAAC
		Reverse	GTCAGCTGGATAGCGACA
Collagen I	Rat	Forward	TGGACATTAGGCGCAGGAA
		Reverse	GAGAGAGCATGACCGATGGATT
Collagen III	Mouse	Forward	ACGTAGATGAATTGGGATGCAG
		Reverse	GGGTTGGGGCAGTCTAGTC
Collagen III	Rat	Forward	CCCAACCCAGAGATCCCATT
		Reverse	GAAGCACAGGAGCAGGTGTAGA
IL-1*β*	Mouse	Forward	GGGCCTCAAAGGAAAGAATC
		Reverse	TACCAGTTGGGGAACTCTGC
IL-6	Mouse	Forward	AGTTGCCTTCTTGGGACTGA
		Reverse	TCCACGATTTCCCAGAGAAC
IL-17	Mouse	Forward	TCCAGAAGGCCCTCAGACTA
		Reverse	AGCATCTTCTCGACCCTGAA
TNF-*α*	Mouse	Forward	CCCAGGGACCTCTCTCTAATC
		Reverse	ATGGGCTACAGGCTTGTCACT
GAPDH	Mouse	Forward	AACTTTGGCATTGTGGAAGG
		Reverse	CACATTGGGGGTAGGAACAC
GAPDH	Rat	Forward	GACATGCCGCCTGGAGAAAC
		Reverse	GACATGCCGCCTGGAGAAAC

**Table 2:** Echocardiographic data of each group.

Groups	Sham+AAV9-shRNA	Sham+AAV9-shPrx5	TAC + AAV9-shRNA	TAC + AAV9-shPrx5
HR (bpm)	523 ± 48	519 ± 39	511 ± 40	516 ± 44
LVEDd (mm)	3.51 ± 0.23	3.63 ± 0.35	4.51 ± 0.36∗	5.32 ± 0.29^**#**^
LVEDs (mm)	1.93 ± 0.16	1.88 ± 0.12	3.18 ± 0.21∗	4.23 ± 0.29^**#**^
LVPWd (mm)	0.66 ± 0.06	0.64 ± 0.05	1.21 ± 0.11∗	1.43 ± 0.13^**#**^
LVPWs (mm)	1.06 ± 0.10	1.05 ± 0.09	1.83 ± 0.13∗	2.33 ± 0.15^**#**^
IVSd (mm)	0.64 ± 0.05	0.63 ± 0.06	1.17 ± 0.09∗	1.46 ± 0.13^**#**^
IVSs (mm)	1.06 ± 0.11	1.04 ± 0.10	1.82 ± 0.15∗	2.24 ± 0.16^**#**^
FS (%)	45.41 ± 2.68	46.53 ± 2.81	29.76 ± 3.03∗	20.34 ± 2.54^**#**^

*n* = 8 for each group. ^∗^*P* < 0.05 vs. the sham group; ^#^*P* < 0.05 vs. the TAC group.

## Data Availability

We declare that the materials described in the manuscript, including all relevant raw data, will be freely available to any scientist wishing to use them for noncommercial purposes, without breaching participant confidentiality.
